# Supplementary material for: Lysine-selective molecular tweezers are cell penetrant and concentrate in lysosomes
Source: Commun Biol. 2021 Sep 14;4:1076. doi: 10.1038/s42003-021-02603-2 (PMC8440717; doi:10.1038/s42003-021-02603-2)
Supplement: Supplementary file 2 — Supplementary Information [file 42003_2021_2603_MOESM2_ESM.pdf]

Supplementary Information

Lysine-selective molecular tweezers are cell-penetrant and concentrate in lysosomes

Zizheng Li[1], Ibrar Siddique[1], Inesa Hadrović[2], Abbna Kirupakaran[2], Jiwen Li[3], Ye Zhang[3,4,5], Frank-Gerrit Klärner[2], Thomas Schrader[2], and Gal Bitan*[1,4,5]

[1]Department of Neurology, David Geffen School of Medicine, University of California at Los Angeles, 635 Charles E. Young Drive South, Los Angeles, CA 90095, USA

[2]Institute of Chemistry, University of Duisburg-Essen, Universitätsstr. 7, 45117 Essen, Germany

[3]Department of Psychiatry and Biobehavioral Sciences, David Geffen School of Medicine, University of California at Los Angeles, 635 Charles E. Young Drive South, Los Angeles, CA 90095, USA

[4]Brain Research Institute, University of California at Los Angeles, Los Angeles, CA 90095, USA

[5]Molecular biology Institute, University of California at Los Angeles, Los Angeles, CA 90095, USA

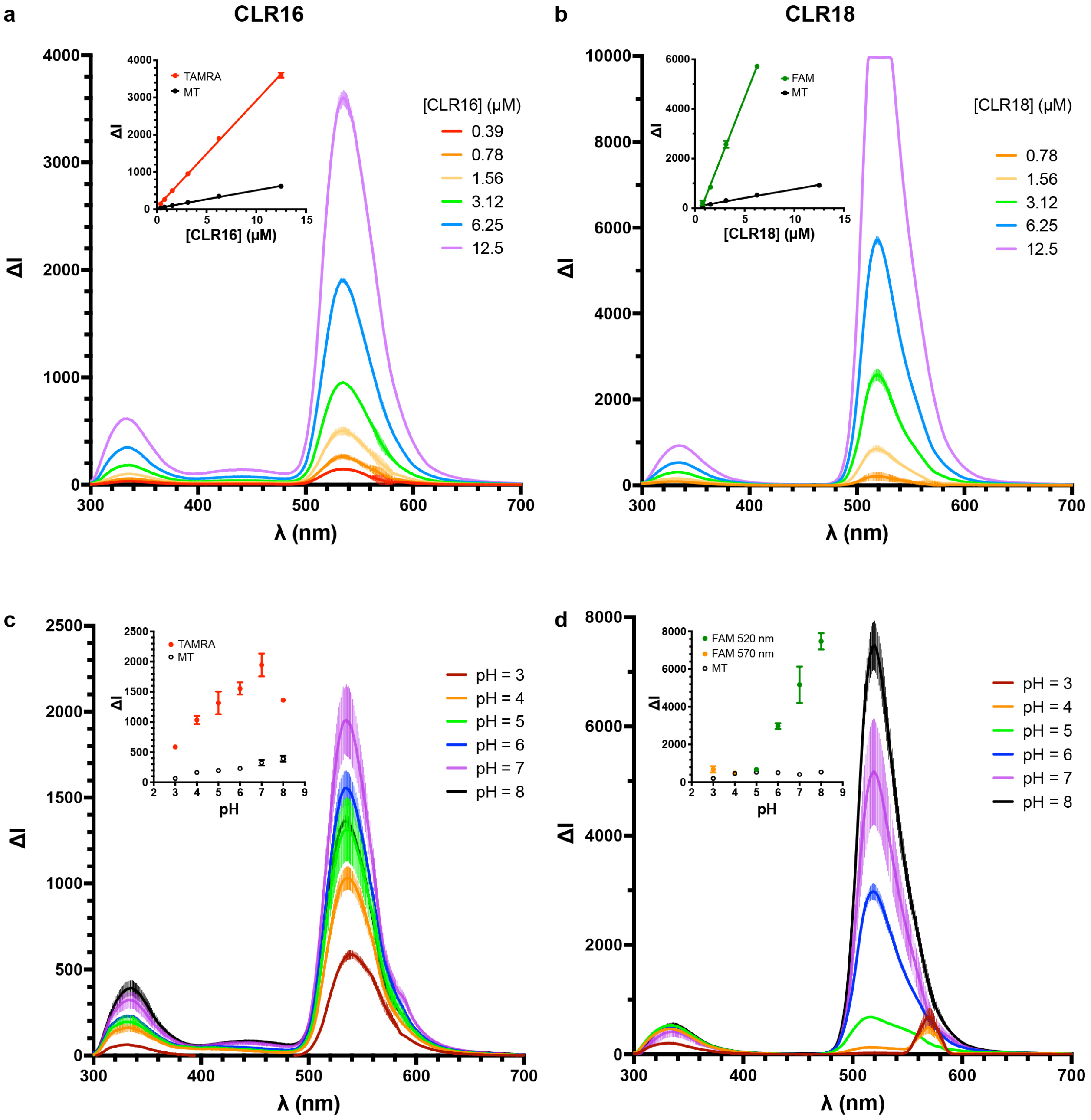

Supplementary Figure 1. Concentration- and pH-dependence of CLR16 and CLR18's fluorescence. a, b) CLR16 (a) or CLR18 (b) were dissolved in PBS at the indicated concentrations and the fluorescence spectra were recorded between 300 and 700 nm using a fluorometer (N = 3 independent experiments). Insets show the concentration dependence of maximal fluorescence of the fluorescent tag, TAMRA at 535 nm in panel a and FAM at 520 nm in panel b, and the MT moiety at ~335 nm. c, d) CLR16 (c) or CLR18 (d) were dissolved at 5 μM in a phosphate-citrate buffer at the indicated pH and the fluorescence spectra were recorded between 300 and 700 nm (N = 3 independent experiments). Insets show the pH dependence of the different peaks.

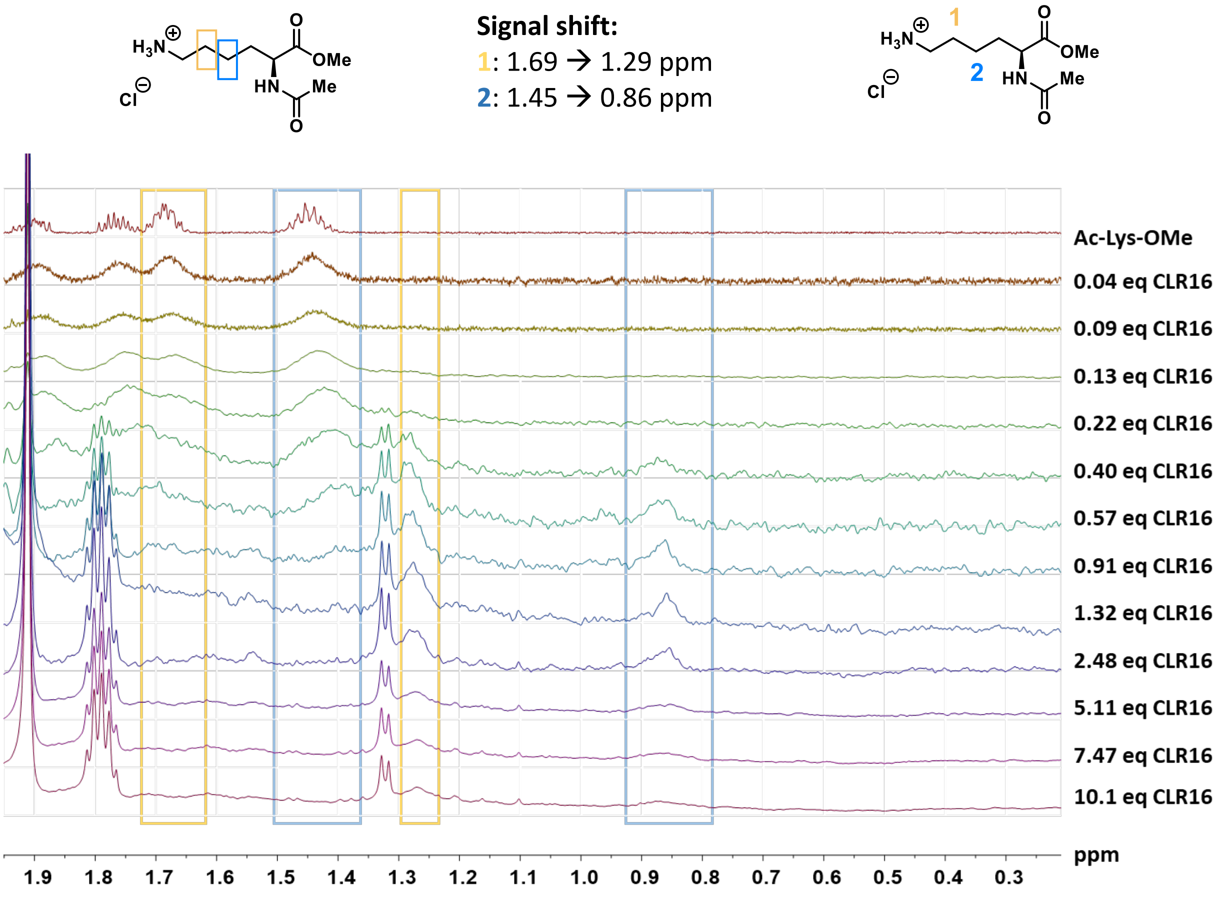

Supplementary Figure 2. NMR titration of Ac-Lys-OMe with CLR16 in 10 mM phosphate buffer (pH 7.4). 0.38 mM Ac-Lys-OMe was titrated with CLR16 (0–3.87 mM). The stacked plot of ^{1}H NMR spectra show separate sets of signals for the alkyl protons 1 and 2 of the lysine side chain in their free and bound state. The yellow and blue boxes correspond to the internal CH_2 group **1** –**CH_2**–CH_2–NH_3^+ (yellow) and **2** –**CH_2**–CH_2–CH_2–NH_3^+ (blue), respectively.

CLR16 (µM)	Ac-Lys-OMe (µM)	Equivalents Ac-Lys-OMe	Intensity I	Δ I
3.32	0.00	0.00	1739.8	0.0
3.32	2.29	0.69	1327.4	412.0
3.32	4.56	1.38	1295.2	444.0
3.32	6.83	2.06	1281.9	458.0
3.32	9.08	2.74	1246.5	493.0
3.32	11.3	3.41	1291.5	448.0
3.32	22.3	6.73	1290.1	449.0
3.32	43.4	13.10	1229.8	510.0
3.32	63.4	19.13	1138.4	601.0
3.32	82.4	24.85	1168.5	571.0
3.32	100.0	30.29	1153.7	586.0
3.32	179.0	53.84	1051.1	688.0
3.32	241.0	72.69	994.2	745.0
3.32	292.0	88.11	1011.7	728.0

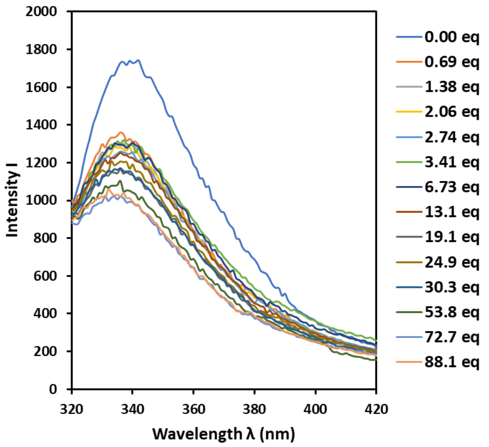

Emission spectra for CLR16 (λ_{ex} = 284 nm, λ_{em} = 339 nm) during titration with Ac-Lys-OMe (sodium phosphate, pH 7.4).

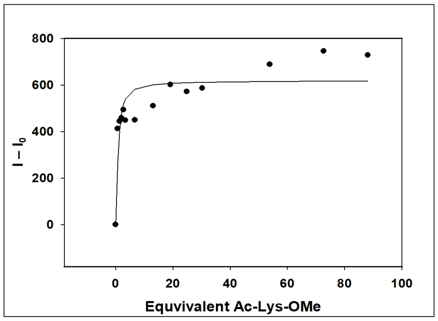

Binding isotherm full titration

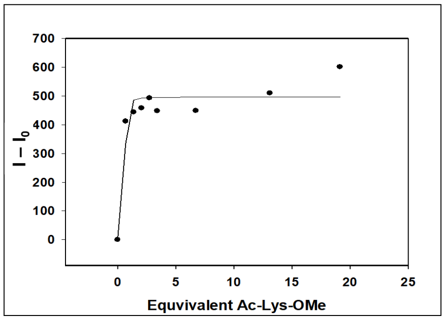

Binding isotherm (0-20 eq. guest)

2:1 Stoichiometry:
Each tweezer binds 2 Lys

First step: K_D = 1.3 nM

Parameter	Value	StdErr	CV(%)
K_A	8.05E+08	4.60E+08	5.72E+04
db	6.19E+05	3.52E+04	5.69E+03

Supplementary Figure 3. Fluorescence titration of CLR16 with Ac-Lys-OMe. CLR16 (3.32 µM) was titrated with Ac-Lys-OMe (0–292 µM). Excitation of the inherent tweezer fluorescence occurred at 284 nm; tweezer emission was recorded at 339 nm. The substantial fluorescence quenching was plotted as binding isotherms and evaluated by nonlinear regression. Best fits were obtained at a 2:1 (Lys/tweezer) stoichiometry. The biphasic behaviour indicates a first very strong binding step, followed by weak association with another Ac-Lys-OMe molecule.

CLR18 (µM)	Ac-Lys-OMe (µM)	Equivalients Ac-Lys-OMe	Intensity I	Δ I
7.76	0.00	0.00	11239.5	0.0
7.76	1.15	0.07	10366.4	873.0
7.76	2.30	0.15	9889.7	1349.8
7.76	3.44	0.22	9789.7	1449.7
7.76	13.7	0.89	9257.7	1981.8
7.76	18.2	1.18	8359.6	2879.9
7.76	22.7	1.47	8313.1	2926.3
7.76	33.8	2.19	8271.2	2968.3
7.76	44.75	2.90	8075.7	3163.7
7.76	87.0	5.65	7883.4	3356.1
7.76	127.0	8.25	7843.7	3395.8
7.76	146.0	9.50	7816.8	3422.6
7.76	165.0	10.72	7786.2	3453.2
7.76	252.0	16.37	7407.2	3832.3
7.76	329.0	21.38	6980.6	4258.8
7.76	398.0	25.85	6876.9	4362.6
7.76	516.0	33.49	6376.8	4862.7
7.76	612.0	39.78	6140.5	5099.0
7.76	693.0	45.04	5919.3	5320.2
7.76	762.0	49.52	5824.6	5414.8

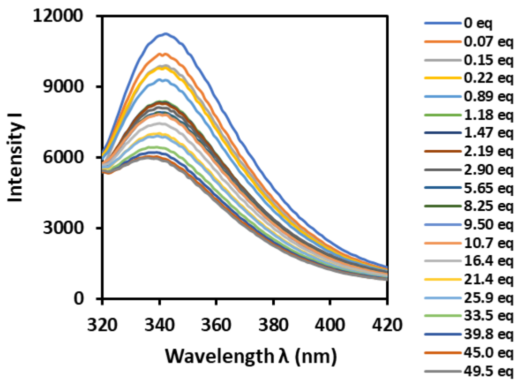

Emission spectra for CLR18 (λ_{ex} = 284 nm, λ_{em} = 342 nm) during titration with Ac-Lys-OMe (sodium phosphate, pH 7.4).

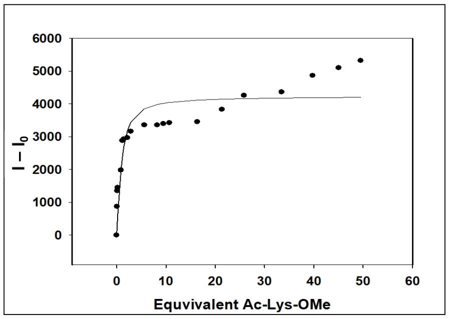

Binding isotherm full titration

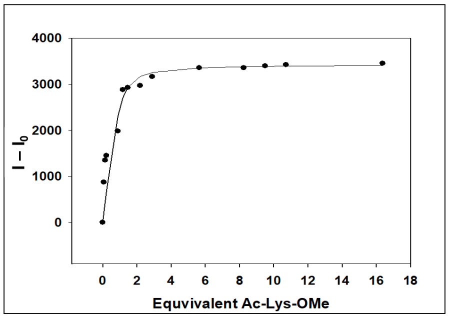

Binding isotherm (0-20 eq. guest)

2:1 Stoichiometry:
Each tweezer binds 2 Lys

First step: K_D = 3.7 nM

Parameter	Value	StdErr	CV(%)
K_A	2.73E+08	1.30E+08	4.75E+04
db	4.24E+06	2.25E+05	5.30E+03

Supplementary Figure 4. Fluorescence titration of CLR18 with Ac-Lys-OMe. CLR18 (7.76 µM) was titrated with Ac-Lys-OMe (0–762 µM). Excitation of the inherent tweezer fluorescence occurred at 284 nm; tweezer emission was recorded at 342 nm. The substantial fluorescence quenching was plotted as binding isotherms and evaluated by nonlinear regression. Best fits were obtained at a 2:1 (Lys/tweezer) stoichiometry. The biphasic behaviour indicates a first very strong binding step, followed by weak association with another Ac-Lys-OMe molecule.

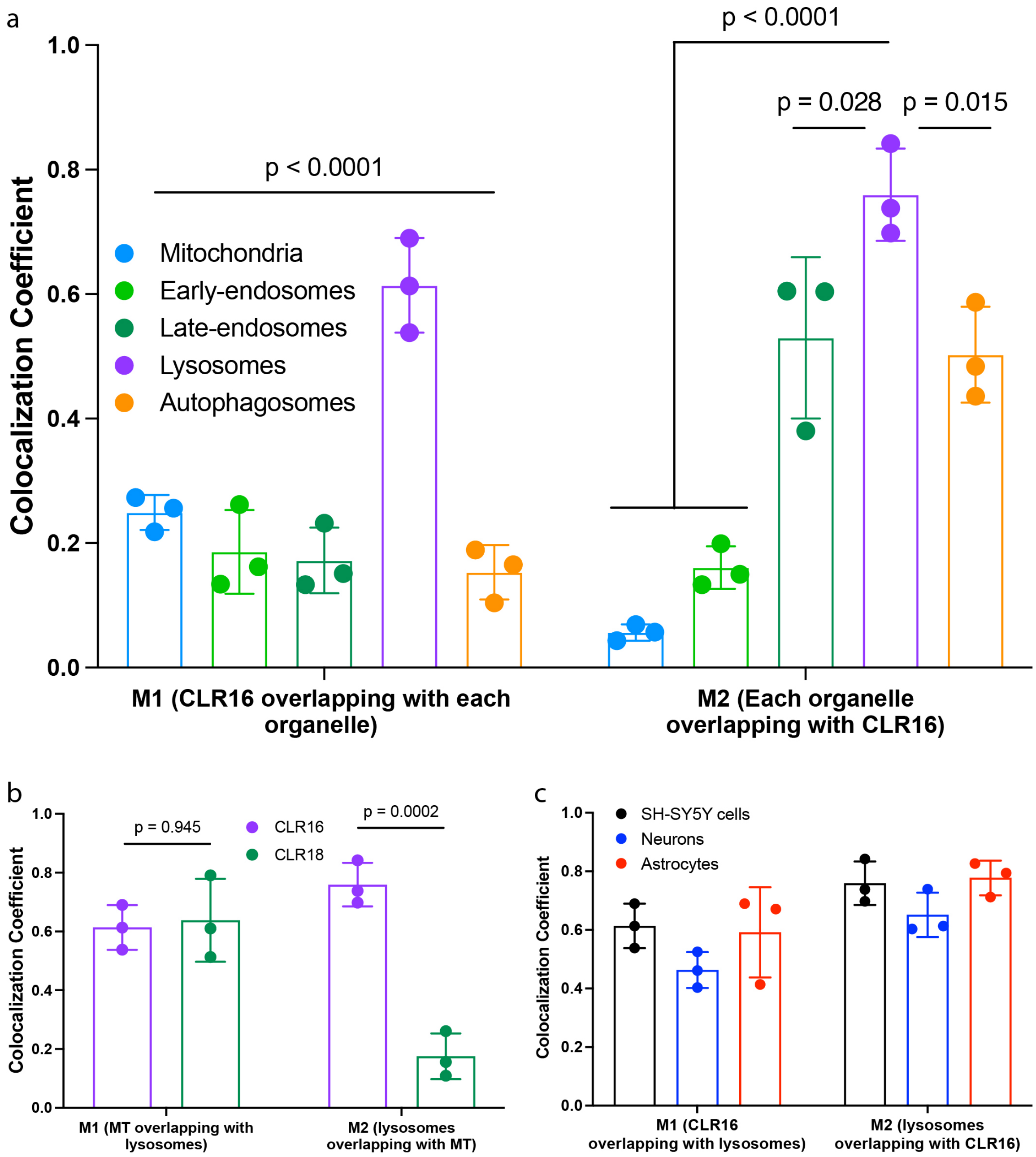

Supplementary Figure 5. Quantitative analysis of the colocalization of MTs with different organelles. The colocalization of MT puncta with the organelles was quantified according to Manders et al.[1] (N = 3 arbitrarily chosen fields of view in the same experiment). The data are shown as mean ± SD. a) SH-SY5Y cells were incubated with 5 µM of CLR16 for 24 h in the presence of specific markers for the different organelles. P-values were calculated by a one-way ANOVA for M1 and M2 separately and are shown only for the comparison of each organelle with lysosomes. b) SH-SY5Y cells were incubated with 5 µM of CLR16 or 10 µM of CLR18 for 24 h in the presence of lysotracker. P-values were calculated by a two-way ANOVA. c) SH-SY5Y cells, primary mouse hippocampal neurons, or primary mouse astrocytes were incubated with 5 µM of CLR16 for 24 h in the presence of lysotracker. P-values were calculated by a two-way ANOVA and were in the range 0.1735–0.9927 (not shown in the figure).

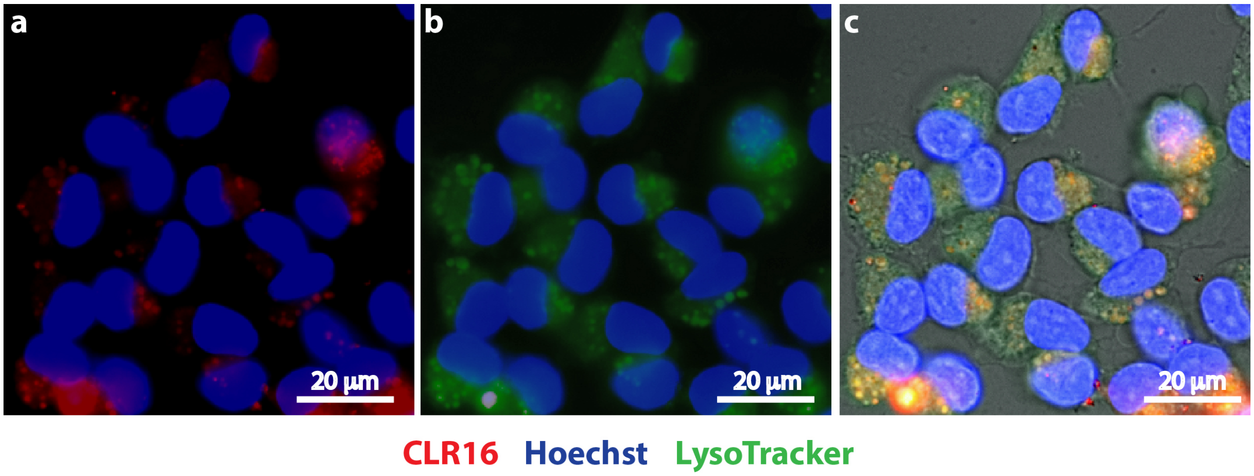

Supplementary Figure 6. CLR16 is internalized into astrocytes and colocalizes with lysosomes. Primary mouse astrocytes were obtained by immunopanning a) The cells were incubated with 5 µM CLR16 for 12 h and nuclei were stained with Hoechst. b) Lysosomes were stained with LysoTracker™ (pseudo-colored green). c) Overlap of panels a and b shows colocalization of CLR16 with lysosomes. The cell morphology is shown by overlap with the brightfield image.

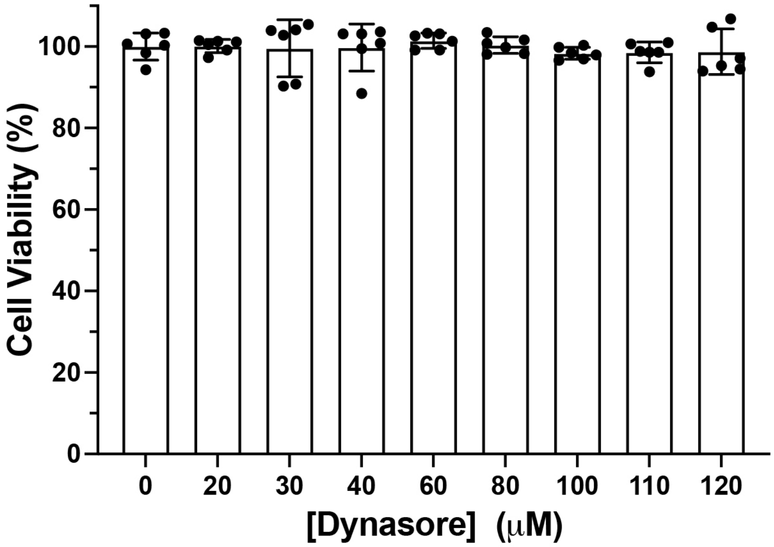

Supplementary Figure 7. Dynasore is not cytotoxic under the experimental conditions used. SH-SY5Y cells were incubated with 5 µM CLR16 and Dynasore at the indicated concentrations for 6 h. Cell viability was measured using the PrestoBlue Assay according to the manufacturer's protocol (N = 6 wells in one experiment). The data are presented as mean ± SD.

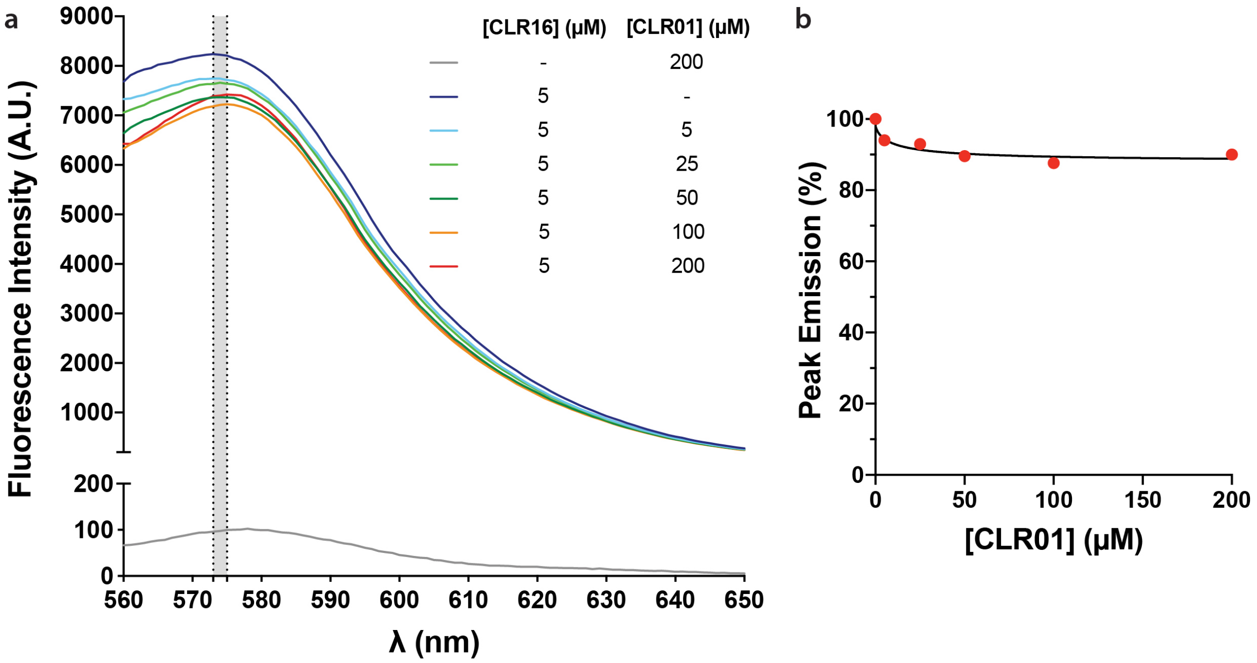

Supplementary Figure 8. CLR01 quenches CLR16 fluorescence weakly. a) 5 µM CLR16 was mixed with different concentrations of CLR01 in DMEM cell-culture medium and the fluorescence was measured between 560 and 650 nm. b) The maximum fluorescence, indicated by the gray area in panel A was plotted against CLR01 concentration.

A

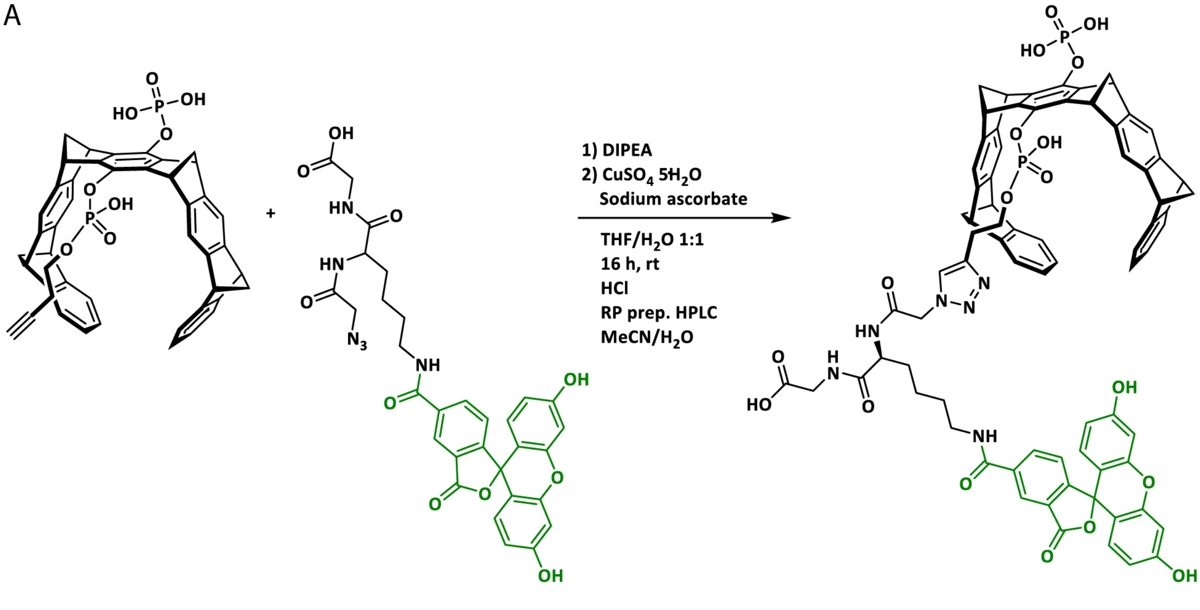

1 2 CLR18

B

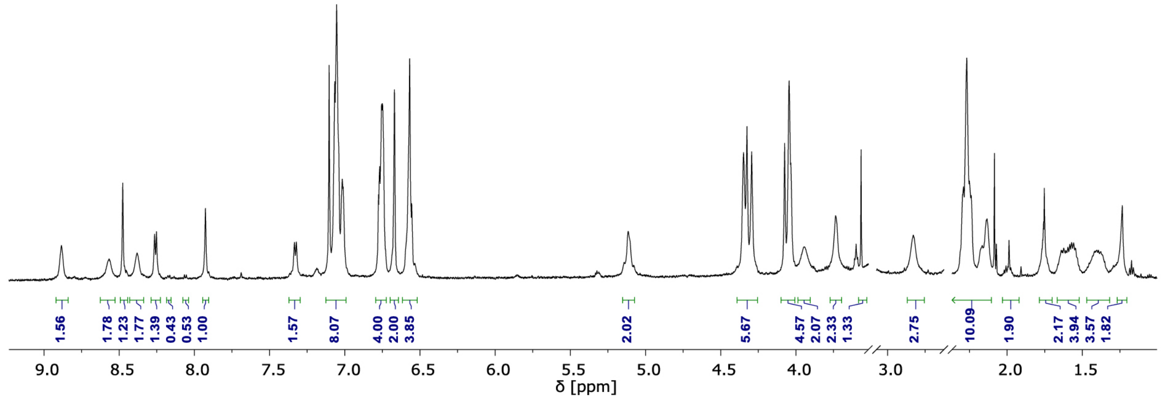

Supplementary Figure 9. Preparation of CLR18. a) Schematic of the reaction between the MT precursor **1** and FAM-azide **2** to yield CLR18. b) ^{1}H-NMR spectrum of the final product.

Supplementary Reference

1 Manders, E. M. M., Verbeek, F. J. & Aten, J. A. Measurement of Colocalization of Objects in Dual-Color Confocal Images. *J Microsc-Oxford* **169**, 375-382, doi:DOI 10.1111/j.1365-2818.1993.tb03313.x (1993).
