# Supplementary material for: Lysine-selective molecular tweezers are cell penetrant and concentrate in lysosomes
Source: Commun Biol. 2021 Sep 14;4:1076. doi: 10.1038/s42003-021-02603-2 (PMC8440717; doi:10.1038/s42003-021-02603-2)
Supplement: Supplementary file 3 — Description of Additional Supplementary Files [file 42003_2021_2603_MOESM3_ESM.pdf]

Description of Additional Supplementary Files

File name: Supplementary Movie 1.

Description: SH-SY5Y cells were incubated with 5 µM CLR16 for 18 h, washed to remove CLR16 from the medium, and imaged by light and fluorescence microscopy. The 3D projection movie was created by combining Z-stacked images using the 3-D image viewer of BZ-X analyzer software.

File name: Supplementary Movie 2.

Description: SH-SY5Y cells were transiently transfected with GFP-actin, incubated with 5 µM CLR16 for 18 h, nuclei were stained with Hoechst, and lysosomes with LysoTracker™ (pseudo-colored cyan). The 3D projection movie was created by combining Z-stacked images using the 3-D image viewer of BZ-X analyzer software. Similar to Figure 4c, the diffuse red fluorescence of CLR16 is seen in the cell culture medium, whereas the movie shows that inside the cells nearly every cyan and red punctum overlap, demonstrating the colocalization of CLR16 with the lysosomes.

File name: Supplementary Data 1.

Description: Source data for all the graphs.

Fig. 5 source data – Inhibition of CLR16 internalization by Dynasore.

Fig. 6 source data – Time-dependent fluorescence in different cellular compartments.

Fig. 9 source data – Inhibition of CLR16 internalization by CLR01.

Supplementary Figure 1 source data – Concentration and pH fluorescence titrations.

Supplementary Figure 3 source data – Fluorescence titration of CLR16 with Ac-Lys-OMe.

Supplementary Figure 4 source data – Fluorescence titration of CLR18 with Ac-Lys-OMe.

Supplementary Figure 5 source data – Quantitation of colocalization.

Supplementary Figure 7 source data – Dyansore cytotoxicity.

Supplementary Figure 8 source data – CLR01 quenching of CLR16 fluorescence.
