# Supplementary material for: Lysine-selective molecular tweezers are cell penetrant and concentrate in lysosomes
Source: Commun Biol. 2021 Sep 14;4:1076. doi: 10.1038/s42003-021-02603-2 (PMC8440717; doi:10.1038/s42003-021-02603-2)
Supplement: Supplementary file 7 — Reporting Summary [file 42003_2021_2603_MOESM7_ESM.pdf]

nature portfolio

Corresponding author(s): Gal Bitan

Last updated by author(s): Aug 10, 2021

Reporting Summary

Nature Portfolio wishes to improve the reproducibility of the work that we publish. This form provides structure for consistency and transparency in reporting. For further information on Nature Portfolio policies, see our Editorial Policies and the Editorial Policy Checklist.

Statistics

For all statistical analyses, confirm that the following items are present in the figure legend, table legend, main text, or Methods section.

n/a	Confirmed	
☐	☒	The exact sample size (n) for each experimental group/condition, given as a discrete number and unit of measurement
☐	☒	A statement on whether measurements were taken from distinct samples or whether the same sample was measured repeatedly
☐	☒	The statistical test(s) used AND whether they are one- or two-sided<br>*Only common tests should be described solely by name; describe more complex techniques in the Methods section.*
☒	☐	A description of all covariates tested
☒	☐	A description of any assumptions or corrections, such as tests of normality and adjustment for multiple comparisons
☐	☒	A full description of the statistical parameters including central tendency (e.g. means) or other basic estimates (e.g. regression coefficient) AND variation (e.g. standard deviation) or associated estimates of uncertainty (e.g. confidence intervals)
☐	☒	For null hypothesis testing, the test statistic (e.g. F, t, r) with confidence intervals, effect sizes, degrees of freedom and P value noted<br>*Give P values as exact values whenever suitable.*
☒	☐	For Bayesian analysis, information on the choice of priors and Markov chain Monte Carlo settings
☒	☐	For hierarchical and complex designs, identification of the appropriate level for tests and full reporting of outcomes
☒	☐	Estimates of effect sizes (e.g. Cohen's d, Pearson's r), indicating how they were calculated

Our web collection on statistics for biologists contains articles on many of the points above.

Software and code

Policy information about availability of computer code

Data collection	Microscopy: BZ-X Viewer,<br>Fluorescence Spectrophotometer: FL Solutions
Data analysis	Image analysis: ImageJ 1.52d, BZ-X Analyzer,<br>Statistical analysis: Graphpad Prism 9

For manuscripts utilizing custom algorithms or software that are central to the research but not yet described in published literature, software must be made available to editors and reviewers. We strongly encourage code deposition in a community repository (e.g. GitHub). See the Nature Portfolio guidelines for submitting code & software for further information.

Data

Policy information about availability of data

All manuscripts must include a data availability statement. This statement should provide the following information, where applicable:

- Accession codes, unique identifiers, or web links for publicly available datasets
- A description of any restrictions on data availability
- For clinical datasets or third party data, please ensure that the statement adheres to our policy

Source data for all the graphs included in this paper are available as Supplementary Data in Excel format. All other data are available from the corresponding author upon reasonable request.

Field-specific reporting

Please select the one below that is the best fit for your research. If you are not sure, read the appropriate sections before making your selection.

☒ Life sciences ☐ Behavioural & social sciences ☐ Ecological, evolutionary & environmental sciences

For a reference copy of the document with all sections, see nature.com/documents/nr-reporting-summary-flat.pdf

Life sciences study design

All studies must disclose on these points even when the disclosure is negative.

Sample size	For microscopy image analysis, measurements from three fields of view in each experiment were used for statistical analysis.
Data exclusions	No data were excluded.
Replication	All experiments were successfully reproduced a minimum of three times.
Randomization	For microscopy image analysis, three fields of view were randomly chosen. Representative images were chosen for the figures.
Blinding	Experimentors were not blinded

Reporting for specific materials, systems and methods

We require information from authors about some types of materials, experimental systems and methods used in many studies. Here, indicate whether each material, system or method listed is relevant to your study. If you are not sure if a list item applies to your research, read the appropriate section before selecting a response.

Materials & experimental systems

n/a	Involved in the study	
☒	☐	Antibodies
☐	☒	Eukaryotic cell lines
☒	☐	Palaeontology and archaeology
☐	☒	Animals and other organisms
☒	☐	Human research participants
☒	☐	Clinical data
☒	☐	Dual use research of concern

Methods

n/a	Involved in the study	
☒	☐	ChIP-seq
☒	☐	Flow cytometry
☒	☐	MRI-based neuroimaging

Eukaryotic cell lines

Policy information about cell lines

Cell line source(s)	SH-SY5Y and HEK293 cell lines stably expressing Tau repeat domain containing the substitution P301S and conjugated to EYFP , referred to as “biosensor cells,” were purchased from American Type Culture Collection (ATCC).
Authentication	Cell lines were used as is and were not authenticated.
Mycoplasma contamination	Mycoplasma decontamination has been carried out occasionally in our laboratory whenever there was a reason to suspect contamination. The cell lines were not tested for mycoplasma contamination.
Commonly misidentified lines (See ICLAC register)	No commonly misidentified cell lines were used.

Animals and other organisms

Policy information about studies involving animals; ARRIVE guidelines recommended for reporting animal research

Laboratory animals	C57BL6 mice were dissected at postnatal day 2 for generation of primary astrocytes. PS19 mice (B6;C3-Tg(Prnp-MAPT*P301S)PS19Vle/J) were purchased from The Jackson Laboratory (stock# 008169). The homozygous transgenic mice were sacrificed at the age of 8 months to obtain brain extract, which were used for the tau seeding experiments. Wild-type littermates from the same line were used at postnatal day 1 for generation of primary hippocampal neurons.
Wild animals	Wild animals were not included in the study.

Field-collected samples	Field-collected samples were not included in the study.
Ethics oversight	All the experiments were carried out in accordance with National Research Council Guide for the Care and Use of Laboratory Animals, approved by the University of California-Los Angeles Institutional Animal Care Use Committee, and performed with strict adherence to the guidelines set out in the National Institutes of Health Guide for the Care and Use of Laboratory Animals.

Note that full information on the approval of the study protocol must also be provided in the manuscript.
